# An Eye Tracking Investigation of Developmental Change in Bottom-up Attention Orienting to Faces in Cluttered Natural Scenes

**DOI:** 10.1371/journal.pone.0085701

**Published:** 2014-01-22

**Authors:** Dima Amso, Sara Haas, Julie Markant

**Affiliations:** Department of Cognitive, Linguistic, & Psychological Sciences, Brown University, Providence, Rhode Island, United States of America; University of Akron, United States of America

## Abstract

This study examined the contribution of visual salience to bottom-up attention orienting to faces in cluttered natural scenes across development. We eye tracked participants 4 months to 24 years of age as they freely viewed 16 natural scenes, all of which had faces in them. In half, the face was also the winner-take-all salient area in the display as determined by the MATLAB SaliencyToolbox. In the other half, a random location was the winner-take-all salient area in the display and the face was visually *non-salient*. We found that proportion of attended faces, in the first second of scene viewing, improved after the first year. Visually salient faces attracted bottom-up attention orienting more than non-salient faces reliably and robustly only after infancy. Preliminary data indicate that this shift to use of visual salience to guide bottom-up attention orienting after infancy may be a function of stabilization of visual skills. Moreover, sociodemographic factors including number of siblings in the home and family income were agents of developmental change in orienting to faces in cluttered natural scenes in infancy.

## Introduction

This study examined the role of bottom-up visual influences in guiding attention orienting to relevant stimuli in the environment, specifically faces. Faces are an important stimulus with respect to information gathering as early as in infancy. Orienting to faces is critical to learning from social interaction in typical development, and may be impaired in atypical development [1], [2]. As such, understanding the developmental trajectory of orienting to faces in cluttered natural scenes, and the potential bottom-up influences on this trajectory, is useful to both understanding typical development and to preventing negative cascades in atypical development.

‘Bottom-up attention orienting’ refers to a series of mechanisms that work in concert to illicit rapid responses to exogenous and salient properties of stimuli or scenes. The computational framework, first conceptually introduced by Koch & Ullman [3] and inspired by the primate visual system [3]-[8], is as follows. Locations in a visual field that are most visually ‘salient’ attract bottom-up attention orienting mechanisms. Any visual field contains feature maps for color, intensity, orientation, motion, depth etc. that quantify the amount of a visual feature at each location in the field. Locations that differ from their surround are tagged. Individual feature maps are then linearly summed into a topographically organized saliency map and a winner-take-all location with the highest saliency value is identified. Attention orienting is then directed toward this winner-take-all salient location, which will be referred to often simply as ‘salient’ throughout this manuscript, even as others that are less salient compete for resources. The selected location is then suppressed, perhaps through an inhibition of return mechanism, and the next most salient location in the display is attended in succession. This framework is particularly relevant for the first several fixations of scene viewing [3], [4], [6], [9], as top-down information, perhaps task goals or otherwise informative stimuli, subsequently guides attention orienting.

Within this framework, there are at least two mechanisms by which visual attention may impact orienting to faces in cluttered natural scenes. The first is through differences at the level of weighting feature maps for computations of saliency, which is the focus of this experiment and will be elaborated on shortly. The second is through a developing ability to suppress interfering information for efficient salient target item selection. A series of studies have shown that developmental change in visual search skill, which theoretically taps this selection process, correlates with developing object perception [10] and also with orienting and sustaining attention to faces in live-action videos [11]. Computational saliency map models of infant data, specifically on the visual search task used in these studies, have indicated that developmental change in difficult visual search tasks is at the level of efficient competition resolution between scene elements for target selection [12], [13].

However, much less is known about how *visual feature processing*, at any point in development, contributes to bottom-up attention orienting to faces in natural cluttered scenes. This is an important question from a developmental perspective. Implicit in the bottom-up attention orienting framework is the idea that the relative contribution of feature maps to the saliency map depends on the state of the viewer [3], [14]. That is, a colorblind individual would weight the color feature less than a typical observer in the winner-take-all summation and hence identify a different location as salient for bottom-up attention orienting. Similarly, more or less observer visual sensitivity to a particular feature, perhaps as a function of visual developmental change, may also result in different winner-take-all locations for bottom-up attention orienting. Studies of infant color vision showed that three types of cones (L, M, and S) were functional by at least 4 weeks of age [15], and others have shown that infants' red/green channel (L–M cone pathway) could be functional by 2 months of age [16]. Infants are known to be sensitive to orientation as early as 6-8 weeks but cannot identify a figure from a background based on orientation until school age [17]. These developmental changes in visual sensitivity alone may bear on the computation of a winner-take-all saliency map, and ultimately on bottom-up attention orienting guidance across development.

There is strong evidence that visual features play an important role in driving discrimination and identification of faces. Even newborns show a unique sensitivity to faces and face-like stimuli [18], [19], which likely stems from the contrast polarity inherent in the stimulus [18], [20]. Contrast polarity refers to the specific light/dark distinction between facial regions (dark eyes/light face or dark/pupil/light sclera). Pallett & Dobkins [21] showed that developmental improvement in face discrimination from childhood to adolescence was also related to change in luminance contrast sensitivity in the observer. Thus, there is evidence that visually salient aspects of face stimuli are relevant for guiding orienting to faces when presented in isolation.

**Figure 1:**
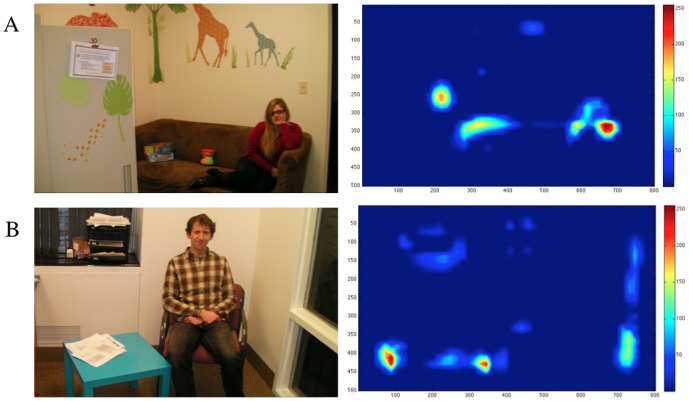
Illustrates images shown to participants and associated saliency heat maps. Hot colors represent higher salience locations. A) Face is winner-take-all most salient area in display as determined by the MATLAB SaliencyToolbox [24]. B) Non-Salient Face example image where faces are not most salient area in the display. Individuals in photographs have given written informed consent, as outlined in the PLOS consent form, to publication of their photograph.

In contrast, this work examined visual influences on orienting to faces in cluttered natural scenes using a combination of eye tracking, area-of-interest, and saliency map algorithm analytic techniques (see also [22]). Using an approach developed in Amso et al. [2], [23], we generated photographs of every day scenes, all of which had faces in them. In half of these, the face was also the most winner-take-all salient area in the display and labeled *salient face* (Figure 1A), as determined by the MATLAB SaliencyToolbox [24]. In the other half, the face was not the most salient area in the scene or *non-salient face* (Figure 1B). This allowed us to parse out the contribution of visual salience in driving initial attention orienting to faces in cluttered natural scenes across development. Based on the review of the literature provided, two outcomes are possible. In one scenario, early attention to faces and face like configurations, on the basis of visual feature information, should result in greater bottom-up attention orienting to salient relative to non-salient faces beginning in early infancy. Alternatively, in light of the computational saliency map framework, rapid developmental change in visual skill in infancy may result in different weighting of visual feature maps, and ultimately winner-take-all saliency maps, in infants as compared to the more stable visual state in young children and adults. In this case, infants would show no preference for salient relative to non-salient faces.

**Figure 2:**
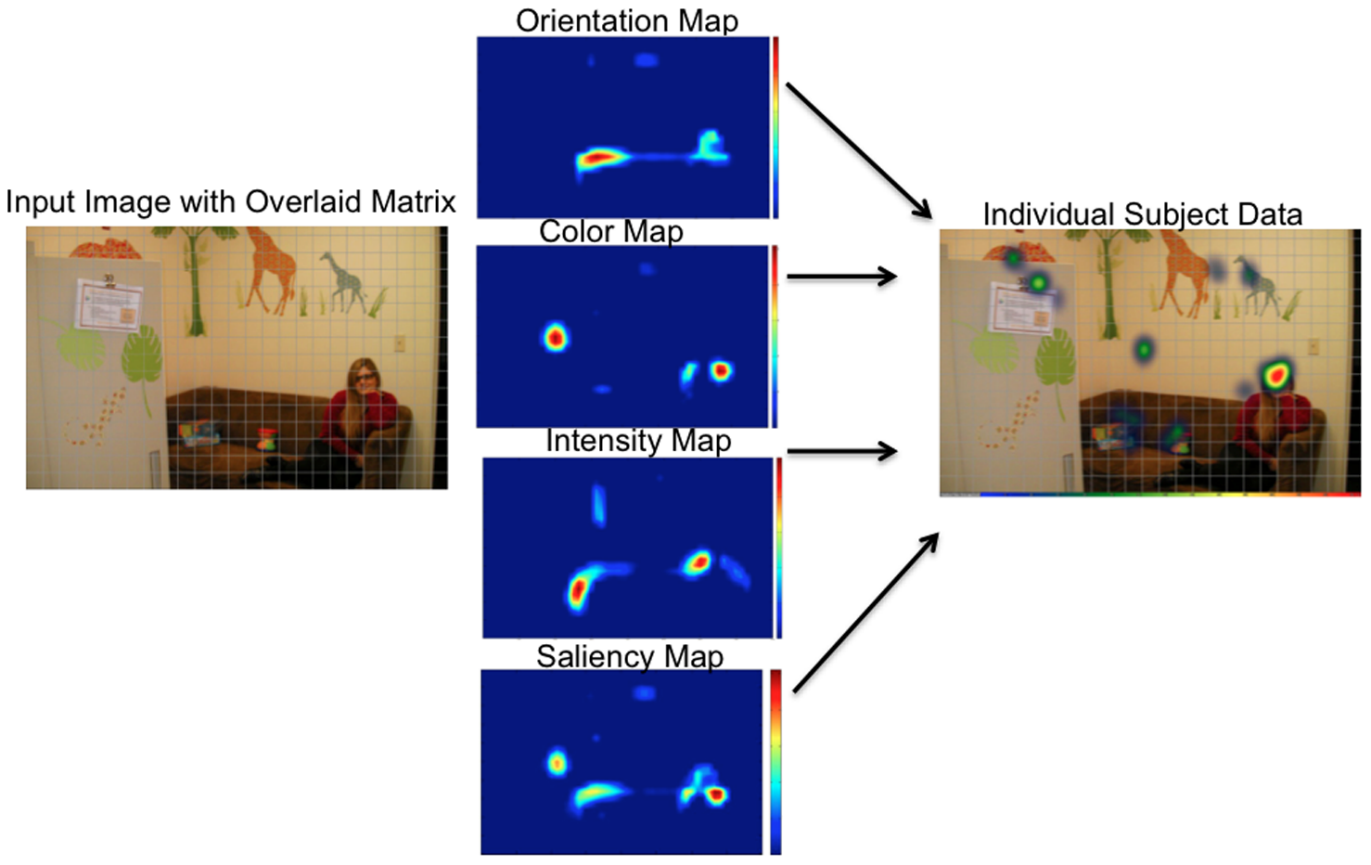
Illustrates the beta coefficient analytic strategy. Each input image was divided into a 16x25 matrix. Saliency and visual feature maps were extracted for each matrix grid location (MGL) and then used as predictors in multiple regression analyses, in order to determine their explanatory power for the duration of looking variable in those MGLs, per participant and image.

In order to test the latter possibility, we have generated an analytic strategy that uses the saliency map algorithm in a novel way. This technique is designed to explore individual and developmental differences in the weighting of color, intensity, and orientation information during natural scene viewing (see also [2]). We generated a matrix grid per image and used the MATLAB Saliency Toolbox [24] to extract color, orientation, intensity, as well as the winner-take-all saliency map values for each matrix grid location (Figure 2). We then extracted duration of looking data per subject for the same matrix grid locations. The extracted Saliency Toolbox visual map values (color, orientation etc.) were then used as predictors for each participant's looking data in individual multiple regressions. Beta coefficients indicating the predictive strength of visual feature maps were then used in group-level analyses to determine differences in how visual feature and combined saliency maps are weighted for attention orienting. The advantage of these beta coefficient values is that they allow for a basic goodness-of-fit between visual feature and saliency maps and participants' free viewing performance across development.

Previous work with this task showed greater preferences for visually salient over non-salient scene locations in 3-5 year-old children diagnosed with ASDs, relative to age and sex-matched typically developing (TD) participants [2]. Specifically, while both groups looked at salient faces within the first several fixations of scene presentation, children with ASDs continued to process the rest of the image consistent with sequential salience while TD children sustained attention to the face. The extent to which children with ASDs used visual salience to guide bottom-up attention orienting in the presence of the face correlated with poorer receptive language and social skills. Therefore, while the focus of our questions is on infancy, we reasoned that mapping the full typical developmental trajectory of orienting to faces in cluttered natural scenes, and specifically the role of visual salience in this process, may be relevant to not only understanding typical development but ultimately to identifying atypical trajectories in infants at-risk for ASDs and older children with the disorder.

## Methods

### Participants

229 participants aged 4 months to 24 years composed the final sample (*M* = 10.3, *SD* = 8.5 years, 117 Female). Of these, *N* = 72 were 4–14 month-old infants (*M* = 7.92, *SD* = 2.88 months, 29 Female). A total of 157 participants were between the ages of 1.5 and 24 years (*M* = 14.7, *SD* = 6.6, 88 Female). Participants were recruited through advertisements and mailings, state department of health birth records, and flyers and brochures in the local community.

### Ethics statement

This study was approved by the Brown University Institutional Review Board. All participants were treated ethically according to the Declaration of Helsinki. Participants and/or legal guardians signed consent documents prior to participation.

### Eye tracking apparatus and measures

Eye movements during free viewing were recorded using a remote eye tracker (SensoMotoric Instruments, SMI, RED system). Participants sat about 70 cm from a 22″ (55.9 cm) monitor. Infants sat in a parent's lap. All calibration and task stimuli were presented using the Experiment Center software native to SMI. Calibration consisted of presenting an attractive, looming stimulus in the upper left and lower right corners of the screen. Presenting the attractive stimulus in the four corners of the screen before the task began validated accuracy.

We used *tracking ratio*, defined as the proportion of time that the eye tracker recorded point of gaze coordinates over the entire task, to further examine quality of data. Participants with <30% tracking ratio contributed very little data and were excluded from analyses (11 infants, *M* = 4.8 months, *SD* = 2.2 months) for a final *N* = 218. The tracking ratio in the excluded group was *M* = 21.8%, *SD* = 10.12%. The tracking ratio in the useable group was *M* = 89.7%, *SD* = 14.8%. Calibration was skewed in three infants who otherwise had high tracking ratios. We therefore coded their proportion of attended faces variable manually, but did not use their data in analyses where the dependent variable could not be hand coded.

### Free Viewing Task

As in Amso et al. [2], participants freely scanned sixteen color photographs (1680×1050 pixels) depicting both indoor and outdoor scenes. Each image was presented for 5 sec, with the order of image presentation randomized across participants. A central fixation target was used to return participants' point of gaze (POG) to the center of the screen between images.

We defined face areas of interest (AOIs) as the face and hair regions combined. The SaliencyToolbox [24] was used to determine the winner-take-all most salient areas in each image. In six images, the face was identified as the number one most visually salient location (Salient Faces, Figure 1A). In six images, the Face AOIs were not most visually salient (Non-Salient Faces, Figure 1B). Our goal was to keep the images as rich with complex and natural statistical structure as possible. As such, perfect control over all image properties across these conditions was not possible. Nonetheless, in selecting images for this analysis, we attempted to include, across conditions, images with similar layout and complexity, that were indoor or outdoor, that had similar size of face (Salient Faces, M = 4%, SD = 3%; Non-Salient Faces, M = 3%, SD = 2%), and whether eyes were looking forward or to the side etc. We were concerned that participants would be clued into looking at faces in the 12 experimental images, as the faces were prominent. Therefore, we included 4 foil imaged, which also contained people, but that were not prominent or central to the scene.

### Data Preprocessing

#### Proportion Attended Face Areas

We were interested in possible differences in bottom-up attention orienting to visually salient versus non-salient faces. Therefore, the analysis for this metric was constrained to the first second of each trial, accounting for the first several fixations of viewing per image [2], [23]. We calculated the proportion of images that each child looked at these Salient and Non-Salient Face areas of interest (AOIs). This was calculated as the proportion of trials in which the participant fixated the AOI type at least once during the 1 sec interval (calculated as number of trials attended/total possible trials). A fixation was defined by SMI's native BeGaze software as 100 ms per 100 pixel dispersion.

#### Beta Coefficients

This analytic strategy used the saliency algorithm (MATLAB SaliencyToolbox [24]) in a novel way. We examined the predictive strength of each color, orientation, intensity, and winner-take-all saliency maps on free viewing. We first generated a down-sampled 16x25 region matrix grid for each image and extracted duration of looking data for each matrix grid location (MGL) in each image per participant, effectively producing a gaze distribution map per participant per image (Figure 2). We then used Saliency Toolbox to extract map values for color, intensity, orientation, and their linear combinations for each location in the 16x25 matrix grid for each image. These conspicuity or ‘feature’ maps delineate MGLs where there is a difference in value from one square to its nearest neighbor along these visual feature dimensions. We then determined the fit of the looking data to both the individual feature maps and the saliency map. Specifically, we used multiple regressions to generate beta coefficients per image and participant for each map. The dependent variable was the participant's duration of looking per MGL in the 16x25 matrix. The predictors were color, orientation, intensity, their individual linear combinations, and the overall winner-take-all saliency maps per MGL.

## Results

### Proportion Attended AOIs

We first asked whether orienting attention to faces in the first several fixations of scene presentation differed as a function of the bottom-up visual salience of the face. A total of *N* = 218 participants contributed data to the analysis. The dependent variable was proportion of attended face AOIs as described above. We conducted a Face Type (Salient × Non-Salient) repeated measures ANCOVA with Age, Average Fixation Count, and Average Saccade Latency, both for the 1 sec interval, per participant modeled as continuous variables. The inclusion of oculomotor variables in this analysis was intended to account for the impact of the variability in these metrics expected both within infants and also in such a wide age range. We found a main effect of Face Type, F(1,214) = 6.15, p = .01. Overall, proportion of orienting to Salient faces (M = 71%, SD = 28%) was higher than to Non-Salient faces (M = 66%, SD = 32%). We also found main effects of Age, F(1,214) = 8.22, p = .005, Fixation Count, F(1,214) = 56.77, p = .000, and Saccade Latency, F(1,214) = 4.11, p<.05 and interactions between Fix Count and Face Type, F(1,214) = 4.67, p<. 05, and Age and Face Type, F(1,214) = 4.95, p<.05. We focused follow-up analyses on the Age × Face Type interaction, as it is relevant to the global goals of this investigation.

**Figure 3:**
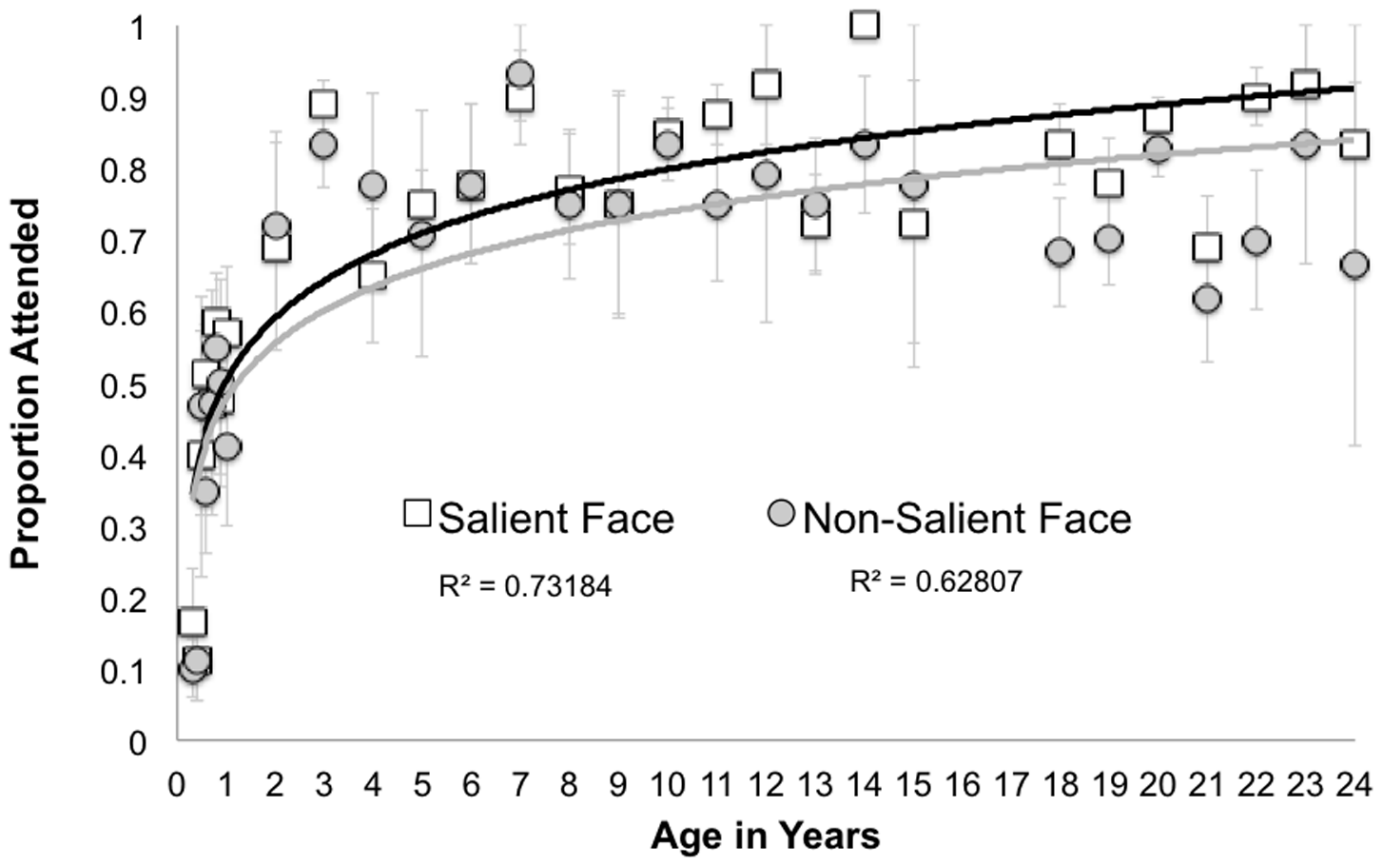
Depicts proportion attended faces in the Salient and Non-Salient Face conditions. Average proportions are binned per month in participants less than one year of age (4 month-olds as separate from 5 month-olds), and per year subsequently (3 year-olds are followed by 4 year-olds). Standard error bars reflect variability within age bin.

Figure 3 shows that this effect of Face Type emerges with Age and specifically is not reliably present during the first year of life. In order to confirm this, we repeated the same ANCOVA only for infants less than 1 year of age (N = 61). This analysis resulted in only main effects of Fixation Count, F(1, 57) = 6.51, p = .01 and Age, F(1,57) = 4.84, p<.05. These data indicate that bottom-up attention orienting to faces increases in infancy, but that greater use of visual saliency to guide attention orienting to social stimuli emerges and increases after the first postnatal year.

### Beta Coefficients

As discussed in the Introduction, one possible reason for infants not showing the bottom-up orienting differential between Salient and Non-Salient faces might be low-level differences in visual skill – the biological state of infants' visual systems. Developmental change in visual skill may result in the system not computing salience in the same way in infancy. We generated the beta coefficients for each color, intensity, and orientation feature maps for all 16 images. These beta coefficients represent the explanatory power of each feature on the distribution of looking per participant. For example, a high intensity beta coefficient means that an infant used intensity to guide attention orienting, while a low intensity coefficients suggests they did not. The values naturally vary with image statistics. For example, color might be dominant in one image and orientation in another. As such, this analysis is only valuable for understanding relative age-related differences in how these values are weighted during free viewing. Finally, we also generated beta coefficients for the winner-take-all saliency map for the 1 sec interval per participant. This was intended to replicate our finding in the previous analysis that the use of salience changes with development.

**Figure 4:**
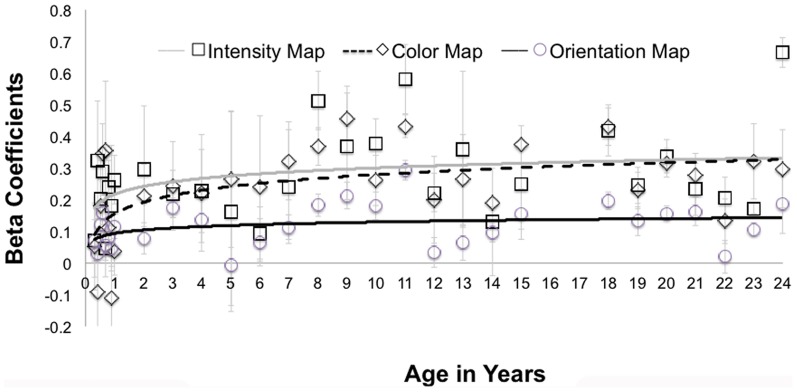
Illustrates beta coefficients for color, intensity, and orientation maps. Average beta coefficients are binned per month in participants less than one year of age, and per year subsequently. Standard error bars reflect variability within age bin.

Multivariate ANCOVA (N = 215) with age modeled as a continuous variable yielded reliable interactions between Age and Color, F(1,213) = 6.98, p<.01, Age and Orientation, F(1,213) = 3.87, p = .05, Age × winner-take-all saliency map beta coefficients, F(1,213) = 4.82, p<.05, and a trending effect for Age × Intensity, F(1,213) = 2.76, p = .09. The overall model for Age was reliable at F(4,210) = 2.50, p<.05. In all cases, as expected from the developmental literature, the use of visual features to guide orienting and of winner-take-all saliency maps increases with age (Figure 4). Excluding infants from this analysis resulted in all interactions with age disappearing (all ps>.05), indicating that these interactions were driven by infants relative to the rest of the sample. Taken together with the proportion attended faces data, these results suggest that visual salience does result in higher probability of getting to a face in cluttered natural scenes, but that it is unlikely to be predominantly driving getting to faces in the first year of life, potentially as a function of rapid change in visual skills.

### Agents of Change in Bottom-up Orienting to Faces in Infancy.

This final analysis is exploratory. Figure 3 and the results above clearly show that proportion attended faces increases throughout infancy and stabilize shortly thereafter. As such, our final analysis considered more broadly agents of developmental change and individual differences in orienting attention to faces in the first several fixations of scene viewing in the infant group. We collapsed across proportion attended Salient and Non-Salient Faces and used this average per participant as the dependent variable in a multiple regression analysis. We modeled oculomotor (average fixation duration, average saccade latency), visual beta coefficients (for color, intensity, orientation) and sociodemographic (family income in dollars, stay-at-home mother status, parental education in years, number of siblings in the home) variables as predictors. We used data from the full trial duration (5 sec) for the oculomotor and visual feature beta coefficients predictors. This was done to ensure the most stable and representative values per infant went into this individual differences analysis. Of 61 infants in the 4 – 14 month-old range, N = 47 had data for all task and demographic variables (of these 42 had reported income, 5 had imputed income using standard procedures, see [25]. All tolerance collinearity statistics were >.2. The model explained a significant proportion of the variance, F(10,36) = 10.66, p = .000, R^2^ = .75. See Table 1 for results. Infant age predicted greater proportion of attended faces in the first second of scene viewing. Interestingly, higher family income and a larger number of siblings in the home also predicted more orienting to faces in infancy within the first second of natural scene viewing. The increasing use of color was a reliable positive predictor of orienting to faces. Finally, oculomotor metrics were relevant to orienting attention to faces across all of our analyses. Table 1 shows that longer fixation durations and slower saccades supported better attention orienting to faces in the model. This indicates that better oculomotor control is an important part of the selection process.

**Table 1:** Predictors of proportion attended faces.

Variable	B *(SE)*
Constant	-.15 (.*25*)
Age	.24* (*0.12*)
Stay At Home Status	.05 *(.05)*
Number of Siblings	.07* (*0.03*)
Parental Education	-.02 (*0.02*)
Family Income	.001* *(.001)*
Fixation Duration	.002** (*0*)
Saccade Latency	0* *(0)*
Color Beta Coefficients	.21 * *(.09)*
Intensity Beta Coefficients	.02 *(.10)*
Orientation Beta Coefficient	.24 *(.21)*
	*R^2^* = .75
	N = 47

*ps<.05.

**p = .000.

### Control Analyses

In order to verify that our effects were specific to bottom-up attention orienting in the first several fixations of scene presentation (or first second as operationalized here), we examined sustained attention to faces for the duration of the 5 sec interval (calculated as a proportion total looking time at face/total looking time at scene). We repeated the Face Type (Salient × Non-Salient) repeated measures ANCOVA with Age, Average Fixation Count, and Average Saccade Latency per participant modeled as continuous variables. The analysis (N = 215) resulted in no main effect of Salience, F(1,211) = .20, p = .66, and no interaction between Face Type and Age, F(1,211) = 1.88, p = .17. This verifies that visual salience plays a part in driving attention orienting to faces only in the first several fixations of scene viewing. Overall proportion of looking at faces increased with Age, as revealed by a main effect of Age, F(1,211) = 16.48, p = .000, again indicating important developmental change in attending to faces. We asked whether bottom-up attention orienting to faces in the first second correlated with duration of looking at the face. Proportion attended faces in the first second of viewing correlated highly with proportional duration of looking at faces in the both Salient, r(215) = .43, p = .000, and Non-Salient conditions, r(215) = .68, p = .000 across the entire sample.

## Discussion

This work adds several important findings to the literature on developmental change in bottom-up attention orienting to faces in cluttered natural scenes. Because we tested a wide age-range, we were able to estimate the developmental trajectory of bottom-up attention orienting to faces (Figure 3). Most relevant to the goals of the project, visually salient faces attracted bottom-up attention orienting more than non-salient faces reliably only after infancy. We provide some preliminary evidence that the lack of this salient/non-salient face attention orienting differential in infants is potentially a function of rapid change in visual skills. In addition, bottom-up attention orienting to faces in infancy does seem to be influenced by sociodemographic factors, including number of siblings in the home and family income. Finally, we were able to verify that orienting to the face in the first several fixations of free viewing correlated with duration of looking at the face for the entire trial interval, lending validity to this metric as potentially relevant to face processing, learning from faces, or both (also see [2]).

While the exact developmental timing of emergence of orienting and sustaining attention to faces is mixed, the general consensus from previous data is that young infants do not reliably orient first fixations to face stimuli in clutter [26], [27]. By 6 months, studies are mixed with respect to whether first fixations are directed to faces in the presence of distractor stimuli [28]-[30]. Our work is unique in that we examined the full developmental trajectory of these effects, from infancy to adulthood. We add to this growing literature the finding that that developmental change in bottom-up attention orienting to faces continues beyond infancy, and is influenced by the visual salience of the face only after infancy (Figure 3).

Critically, stimulus salience does not drive differential bottom-up attention orienting to visually salient faces in infants. This finding is consistent with a recent study from Althaus & Mareschal [22] examining the role of bottom-up saliency in category learning. Consistent with our findings, data from Althaus & Mareschal [22] indicated that bottom-up attention orienting is more driven by saliency early in their task. Also consistent with our findings, they found that the influence of visual salience was greater in 12 month-old infants than in younger 4 month-old infants. The beta coefficient analysis in our work sheds light on their results and our own. Beta coefficients indicated that infants, relative to the rest of the sample, weighted color and orientation reliably less than older groups for attention orienting guidance during free viewing. In addition, the beta coefficient for color was positively predictive of orienting to faces in infants (Table 1). Unlike the full winner-take-all saliency map computation, these beta coefficients are not based in any computational summation or function. They simply represent how much color there is at a location and whether this was predictive of whether participants looked in that location. We take this as cautious support of the interpretation that changes in visual skill of the observer are relevant to calculation of saliency in infancy. This beta coefficient approach is more powerful than simply correlating performance on this task with visual sensitivity on some psychophysical measure. Indeed, it negates the correlation problem inherent in linking performance on one task with that on another. The added advantage here is that we can measure differential weighting of these features against each other in the same scene, and also across development. This approach takes into account both sensitivity and relative use of feature information during free viewing.

To sum up the results thus far, face stimulus salience is unlikely to be uniquely causal with respect to developmental change in attention orienting to faces in infancy. Rather, a combination of developing visual skills and sociodemographic variables were positively predictive of attention orienting to faces, over and above changes in age (Table 1). Longer fixation durations and slower saccade latencies both predicted higher proportions of orienting to faces in infancy, indicating a role for oculomotor control in guiding attention toward a desired stimulus. Greater use of color also played a role in more attention orienting to faces. This does not mean that color processing is important for bottom-up attention orienting to faces per se. Rather, it indicates that color was defining of face regions in our particular stimuli. Hence, infants with higher beta coefficients for color were more likely to orient attention to the faces.

Finally, we found that number of siblings in the home and family income were positively predictive of attention orienting to faces in the first several fixations of scene viewing. For the former, a simple speculation may be that number of children in the home is a variable that indexes mere exposure to faces, which has been shown to be important for complex face processing [31]. For the latter, we defer to a long and rich history in developmental science for examining the role of the caregiving environment in social development and for linking the quality of the caregiving environment to socioeconomic status (e.g., see for review, [32]).

To speculate briefly, family income may influence attention orienting to faces in two, perhaps interactive, ways. The first is by providing regular exposure to faces through enriching opportunities such as music and gym classes and museum visits. Alternatively, or perhaps in addition, sociodemographic factors may support better bottom-up attention orienting to faces in infancy via developmental change in the selective attention mechanism introduced in the introduction. Frank, Amso, & Johnson [11] recently found a relationship between developing visual selective attention skill, as measured by resolving competition for selection in visual search tasks, and orienting and sustaining attention to faces in young infants. Clearfield & Jedd [33] showed that socioeconomic status impacts the development of visual attention in infants and specifically for increasingly complex stimuli. As such sociodemographic factors may be at the heart of driving developmental change in efficient selection via suppression skills, which then are guiding orienting to faces regardless of their visual salience, in our infant sample. This interpretation merits testing in future studies.

We highlight possible limitations of this work. First, this study would benefit from the addition of motion in the service of creating the most naturalistic viewing experience. This addition will be very important in future work. Secondly, we focused only on images that had a social stimulus, as our initial question was focused on the developmental influences on bottom-up attention orienting to faces. As a consequence, we cannot speak to whether these data are relevant to orienting to other object classes.

In sum, visual salience is relevant to bottom-up attention orienting to faces but only after infancy. Amso et al [2] found that 3-5 year-olds with ASDs showed a greater reliance on visual salience for bottom-up attention orienting than matched TD participants, for both social and non-social stimuli. A critical question for future work is whether the emergence of this reliance in infants at-risk for ASDs is earlier than is seen in in TD infants, and whether this may contribute to atypical visual attention in this population.
